# Influence of Preparation Reconstruction on the Compressive Strength of CAD/CAM Ceramic Inlays

**DOI:** 10.1155/2019/7307649

**Published:** 2019-01-01

**Authors:** Bruna Salamoni Sinhori, Luiz Clovis Cardoso Vieira, Luiz Narciso Baratieri

**Affiliations:** ^1^Professor Graduate Curses Operative Dentistry, University of Caxias do Sul, Caxias do Sul, RS, Brazil; ^2^Professor, Undergraduate and Graduate Courses Operative Dentistry, Federal University of Santa Catarina, Florianopolis, SC, Brazil

## Abstract

The aim of this study was to evaluate whether the compressive strength of lithium dissilicate ceramic inlays is influenced by the substrate (dentin or composite resin build-up) and to compare it to nonrestored teeth. Thirty freshly extracted human maxillary third molars were selected and randomly ascribed to three groups (n=10). Standardized Class II MOD preparations were made (bucco-palatal width = 2/3 of the intercuspal width and 2/3 of the width at the tooth equator for the proximal boxes), varying the extension of the preparations (Group 2: preparation limited to tooth structure; Group 3: pulpal floor of the preparation rebuilt with composite resin, IPS Empress Direct, restored with lithium dissilicate CAD/CAM ceramic inlays (e.max CAD) and cemented with a resin cement (Variolink II)). All groups were subjected to compressive strength test (1mm/min crosshead speed). The results showed that the fracture strength of G1 (control group) was significantly higher than G2 and G3, while within these groups there were no statistically significant differences. Both groups restored with lithium dissilicate restorations did not reach the fracture strength of the sound teeth but were statistically equivalent and sufficient to withstand physiologic masticatory forces.

## 1. Introduction

Posterior teeth play a very important role in the masticatory system especially because they support the highest functional loads [1]. For this reason, high-quality materials with properties similar to the tooth to be restored are desirable [2, 3]. Patients seeking treatment for replacement of extensive, unsatisfactory restorations are common in the routine practice [4]. The quantity and quality of the remaining tooth structure should be considered to define the best restorative option for each case, since extensive restorations weaken the remaining tooth structure [5, 6].

Cavities larger than one third of the intercuspal distance imply that an indirect restoration should be chosen. It should be taken into account that the polymerization shrinkage stress of composite resins is between 2 and 3% in volume [7], and large cavities with high C-Factor are a complicating factor. The hybrid layer is constantly challenged by stresses from the polymerization shrinkage, which may lead to increased susceptibility to crack formation and short-term failure of the restoration. Therefore, the best option is to choose indirect restoration using ceramic materials [8, 9].

The principles of tooth preparation such as resistance form, and 8° to 15° taper of the axial walls in the cervical-occlusal direction should be strictly followed to obtain long-term resistance of the restoration. Preparations for ceramic restorations must present rounded internal angles since the presence of sharp angles and edges generate stresses, leading to crack propagation and possible fracture of the restorative material [10].

The occlusal reduction should be between 1.5 mm and 2 mm and the axial reduction should at least be 2 mm. These depths are considered sufficient to obtain proper thickness of the ceramic restoration. This directly influences the restoration resistance to masticatory loads and thermal variations [11, 12].

However, most of the time it is not possible to achieve an ideal preparation due to the loss of tooth structure. In these cases, it may be necessary to partially replace the lost portion to obtain a suitable preparation [13]. This maneuver can be accomplished using materials with similar behavior to dentin, such as composite resins [14].

The reconstruction of the lost dental portion using composite resin is a conservative treatment alternative. It has been questioned whether teeth with a preparation build-up using composite resin behave similarly to teeth without composite build-up when both are restored with lithium disilicate ceramic inlays and submitted to compressive strength. Therefore, the aim of this study was to evaluate the influence of the preparation build-up using composite resin on the compressive strength of human third molars restored with lithium disilicate ceramic inlays. The hypothesis tested was that the preparation build-up using composite resin does not affect the compressive strength of human third molars restored with lithium disilicate ceramic inlays.

## 2. Materials and Methods

After authorization of the Research Ethics Committee of the Federal University of Santa Catarina (#1.025.321), thirty sound human maxillary third molars extracted for reasons not inherent to the present study were selected. All the subjects who donated the teeth signed a consent form, agreeing that their teeth would be used for this research. Only teeth that presented three cusps were used. The teeth were cleaned with pumice (Maquira, Maringá, PR, Brazil) and the remains of the periodontal ligament were removed with a #12 scalpel blade (Solidor, Barueri SP, Brazil) and were stored (0.1% thymol solution, pH=7) for a period up to 3 months before starting the research.

The teeth were inspected with a 3.5 X magnification glass (BIOART, São Carlos SP, Brazil, Batch #445) seeking for alterations such as cracks, gaps or anomalies that could compromise the study.

To facilitate the sealing of the teeth to carry out the preparation and cementation of restorations, the root portion of each tooth was fixed within a PVC ring (2 cm diameter, 2.5 cm length) with acrylic resin. The teeth were placed with the occlusal surface parallel to the base of the PVC tube, with a paralelometer (BIOART, São Carlos, SP, Brazil). Before placement on the base, the tooth was marked 2 mm below the cement-enamel junction with the aid of a periodontal probe and wet tipped pen, fixed with wax in a rod with the cylindrical end attached to the paralelometer. Then a metal base was used to keep the plastic ring static, isolated with petroleum jelly and the base was filled with acrylic resin, until 2 mm below the CEJ [15].

After fixing, all teeth were cleaned again using pumice and water and prepared according to the variables, varying the preparation depth of the pulp wall, except for the control group: Mesial-occlusal-distal (MOD) preparation; gingival walls 1 mm below the cementoenamel junction; Rounded internal angles; 10° to 12° wall taper.

Ceramic inlays (e.max CAD HT, shade A3,5, size C14, Ivoclar Vivadent Schaan, Liechtenstein, batch #R51252) were fabricated for each preparation and cemented using dual cure resin cement (Variolink II, Ivoclar Vivadent Schaan, Liechtenstein, batch # S46585) according to the manufacturer's instructions.

**Figure 1:**
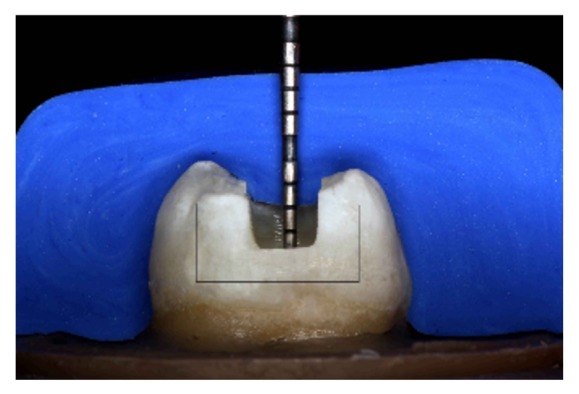
Checking the preparation depth with a periodontal probe to assure that a 2 mm depth was achieved.

Prior to preparation, impressions of the intact crowns were obtained using a PVS impression material (Virtual, Ivoclar Vivadent, Schaan, Liechtenstein, batch #SI4194) to fabricate a silicone index, which would guide the tooth preparation. Thus the preparation depth in the pulp wall was measured from the deepest portion of the sulcus bottom to the pulpal wall (Figure 1).

**Figure 2:**
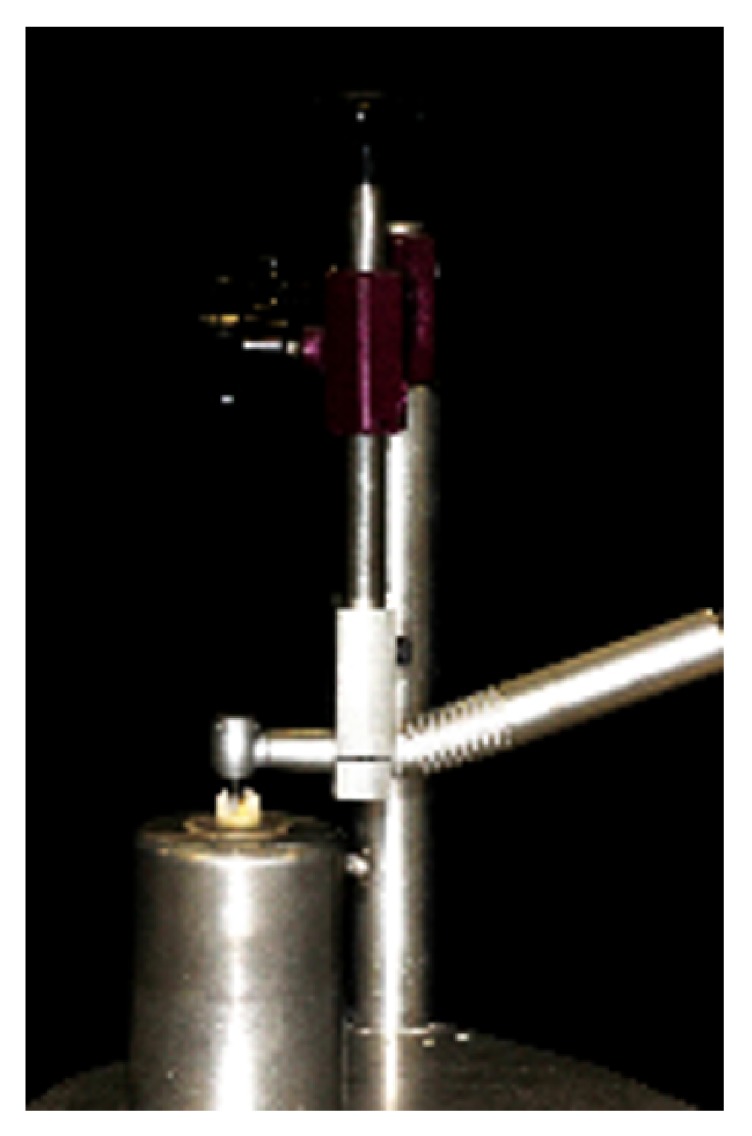
Metal device used to adapt the high-speed hand piece. The goal is to keep the diamond bur parallel to the long axis of the tooth, without slant.

All preparations were performed with rounded-ended conical diamond burs (#3131, KG Sorensen, Cotia, SP, Brazil), replaced each five preparations. The preparations were standardized using a device [16] to attach the high-speed handpiece (KaVo 605C KaVo Joinville, SC, Brazil) to the paralelometer, stabilized in the same position. The long axis of the diamond bur was constant and perpendicular to the occlusal surface of the tooth through the use of this device. Tooth preparation was completed by the same operator through the manual displacement of the tooth embedded in acrylic resin and attached to the metal base (Figure 2)

The intercuspal distance was measured with a digital caliper using and marking the occlusal and proximal surfaces with a wet tip pen. The proximal box was extended approximately 1 mm below the cementoenamel junction from the mesial marginal ridge. The preparation was extended to the buccal and lingual aspects resulting in a similar reduction; e.g., the volume of restorative material was proportional to the size of each tooth. Finishing of the preparation was accomplished using fine grain and extra-fine diamond burs (#3131F and #3131FF, respectively. KG Sorensen, Cotia, SP, Brazil), which were replaced each five teeth.

**Figure 3:**
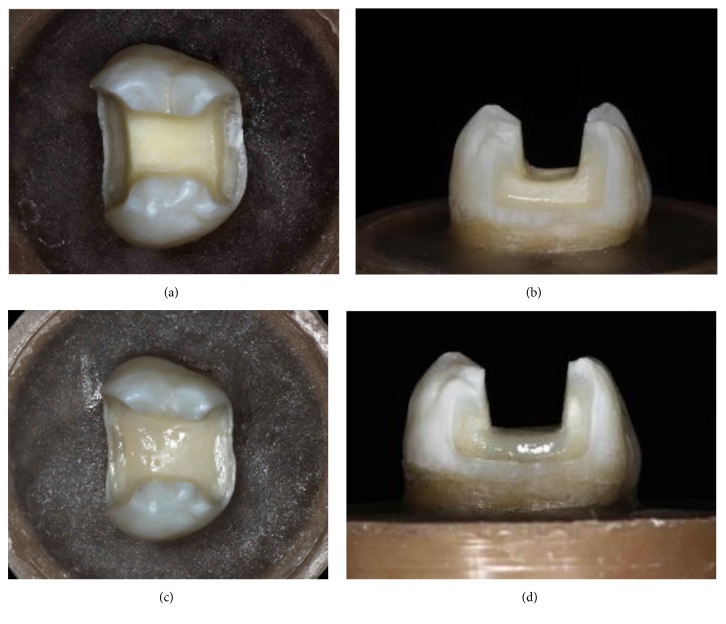
(a) Occlusal view of the preparation of group 1. (b) Proximal view of the finished preparation of group 1. (c) Occlusal view of the preparation of group 2. (d) Proximal view of the finished preparation of group 2.

The teeth were divided according to the type of the preparation as follows: Group 1 (n = 10): control group, sound teeth; Group 2 (n = 10): mesio-occlusodistal (MOD) cavity with isthmus opening of 2/3 of the intercuspal width and 2 mm depth pulpal wall, measured from the sulcus to the pulpal wall. The axial reductions to prepare the proximal boxes were done as 2/3 of the distance from the dental equator in the buccal-lingual direction with 1 mm below the cementoenamel junction, and the distance from the axial-pulpal angle to the gingival wall of 1.5 mm. (Figures 3(a) and 3(b)).

Group 3 (n = 10) is mesio-occlusodistal (MOD) cavity with deep pulpal wall, simulating a tooth with a large carious lesion, isthmus opening equal to 2/3 of the intercuspal distance and 1 mm below the cementoenamel junction. The axial reduction of the proximal boxes was equal to 2/3 of the distance of the dental equator and 1 mm below the cementoenamel junction. Composite resin was used to immediately rebuild the preparation (IPS Empress Direct, shade A2, Ivoclar Vivadent, Schaan, Liechtenstein). Isthmus opening is 2/3 of the intercuspal distance and has 2 mm depth. Proximal boxes are 2/3 of the distance of the dental equator, with 1 mm below the cementoenamel junction and 1.5mm distance from the axial-pulpal angle until the gingival wall (Figures 3(c) and 3(d)).

The depth of the pulpal wall was standardized (2 mm) from the main sulcus to the pulpal wall, with the aid of a previously prepared silicone guide.

To perform the composite resin build-ups, the teeth were etched with 37% phosphoric acid (BM4, Palhoça, SC, Brazil, batch number: 0115/1013) for 15 s in dentin and 30 s in enamel; then a light-cured adhesive system (Tetric N-Bond, Ivoclar Vivadent, Schaan, Liechtenstein, batch # R67742) was applied and light-cured for 20 seconds and they were restored with composite resin (Empress Direct, shade: A2 Dentin, Ivoclar Vivadent, Schaan, Liechtenstein, batch number: S38387). The composite resin was inserted in increments up to 2 mm thickness and light cured for 20 seconds with a LED light-curing unit (Bluephase G2 Ivoclar Vivadent, Schaan, Liechtenstein) (800 mW/cm^2^).

Then, for the cementation of the ceramic inlays restorations, the teeth were etched with 37% phosphoric acid (Power Etching, BM4, Palhoça, Brazil) for 30 seconds on enamel and 15 seconds in dentin and rinsed abundantly with water, and the dentin surface was protected with sterile cotton and the enamel air-dried. The adhesive system (Excite F DSC Ivoclar Vivadent, Schaan, Liechtenstein S36470) was applied to the etched surface and was not light cured until cementation.

**Figure 4:**
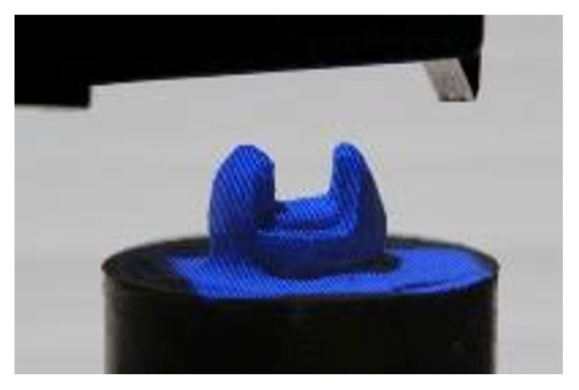
Scanning the preparation with Bluecam device.

**Figure 5:**
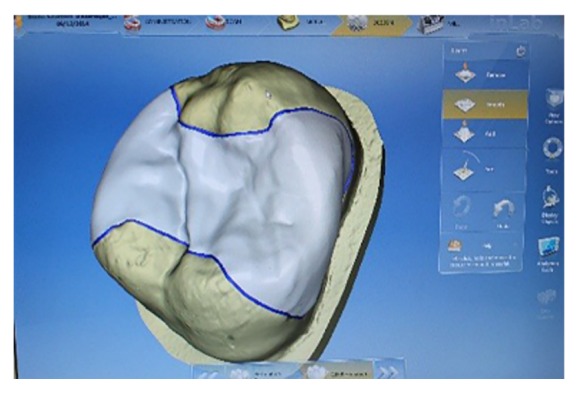
Design stage in Cerec.

Inlays were fabricated using a CAD/CAM technique (Cerec AC, v.4.0, Sirona Dental Systems, Bensheim, Germany). Each preparation was coated with a titanium dioxide spray (OptiSpray, Sirona Dental Systems, Bensheim, Germany, Batch: #2013140338), scanned (Bluecam, Cerec AC,  Sirona, Germany) (Figure 4). For standardization, the original morphology of the restoration was not edited, except for the positioning tools to ensure the correct thickness of the restoration (Figure 5). The blocks used to fabricate the inlays (e.Max CAD, Ivoclar Vivadent, Schaan, Liechtenstein) were crystalized after the milling step in a furnace (P500, Ivoclar Vivadent, Schaan, Liechtenstein) (program P81 for rapid crystallization/Glaze LT, 840°C crystallization temperature).

After the finishing and polishing steps, the ceramic restorations were embedded in a PVS impression material (Virtual, Ivoclar Vivadent, Schaan, Liechtenstein) leaving exposed only the inner surface, which were to be conditioned. The inner surfaces were then etched with a 9% hydrofluoric acid (Porcelain Etch, Ultradent Products, Brazil. Batch: #290414) for 20 seconds, rinsed with water for 20 seconds, cleaned in ultrasonic bath with distilled water for 3 minutes, air-dried, and silane-coated (Monobond Plus, Ivoclar Vivadent, Schaan, Liechtenstein, Batch number: S44734). All restorations were coated with a thin layer of an adhesive system (Excite F DSC, Ivoclar Vivadent, Schaan, Liechtenstein, Batch number: S36470).

**Figure 6:**
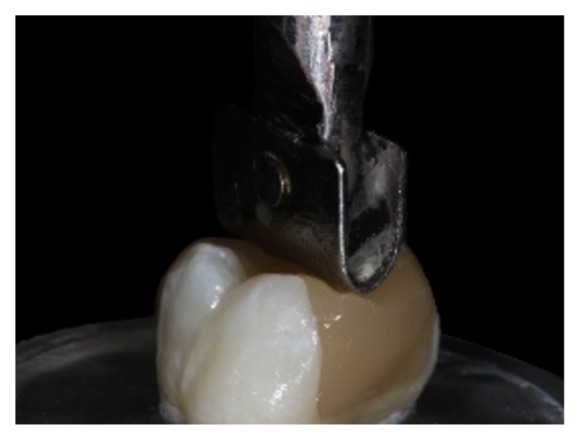
Metal device used to exert constant pressure during the cementation step.

Luting was carried out with a dual-cure resin cement (Variolink II, Ivoclar Vivadent, Schaan, Liechtenstein, Batch: #S411783) following the manufacturer's recommendations. The cement was applied on the preparation and the inner surface of the restoration, followed by light finger pressure toK flow excess material. The specimens were then placed in a device applying a 1 kg force for 2 min [17] to standardize the cement thickness. Excesses were carefully removed with spatula and brushes, and a barrier gel was applied (Liquid Strip Ivoclar Vivadent, Schaan, Liechtenstein: Batch: #S34732) throughout the restoration margins. Light curing was performed from occlusal, mesial and distal aspects using a LED light-curing unit (Bluephase G2 Ivoclar Vivadent, Schaan, Liechtenstein) for 40 seconds on each side. The margins were finished and polished mechanically with abrasive rubbers for composite resin (KG Sorensen, Cotia, SP, Brazil, Batch: #9611) (Figure 6).

Once completed the restorative procedures, the specimens were stored in distilled water at 37°C for 24 hours, to allow water sorption of the resin cement, complete polymerization and achieve maximum compressive strength.

**Figure 7:**
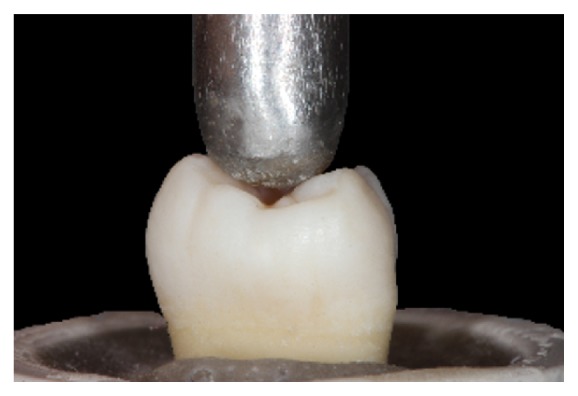
A 6 mm diameter metallic ball touching the tooth of the three cusps.

The compressive strength test was performed in a universal testing machine (EMIC DL200, São José dos Pinhais, PR, Brazil) (TRD 27 load cell, Tesc software, version 3.01, crosshead speed of 1 mm/min). A 6 mm diameter metallic ball, designed to touch the tooth/restoration interface of the three cusps simultaneously was used to accomplish the test (Figure 7).

Data were submitted to one-way analysis of variance (ANOVA) and possible differences detected by the Tukey's post hoc test, both at a significance level of 5% (IBM SPSS Statistics V.21 Armonk, NY, USA).

## 3. Results

Average compressive strength values (N) obtained by the groups, along with their descriptive statistics (standard deviation, minimum and maximum coefficient of variation, and 95% confidence interval) are described in Table 1.

**Figure 8:**
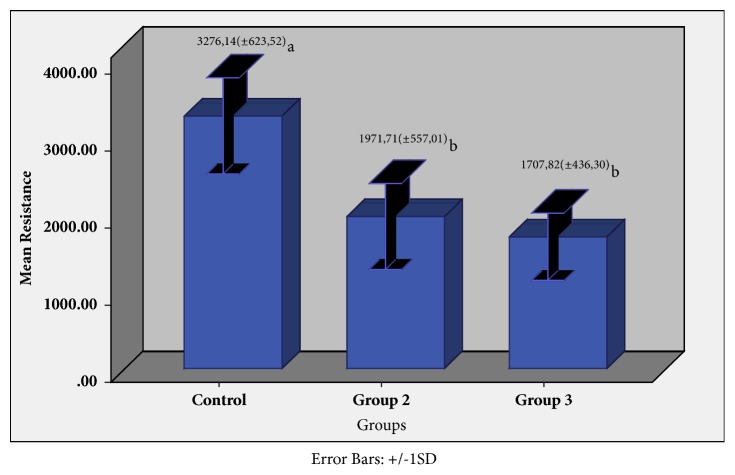
Average resistance to compression values of each group and their respective standard deviations (SD). Averages followed by the same letter indicate no statistically significant differences between groups according to Tukey's post hoc test at a significance level of 0.05.

The compressive strength values show to be normally distributed according to the Shapiro-Wilk normality test (Table 2) and Levine's homogeneous variance statistical test (Table 3). ANOVA detected statistically significant differences between groups (p <0.001) (Table 4). In accordance with the post hoc Tukey's multiple comparison test (Table 5), the compressive strength of the control group was statistically superior to G2 and G3 (p <0.05), while there were no statistically significant differences between the last two (p> 0.05) (Figure 8).

**Figure 9:**
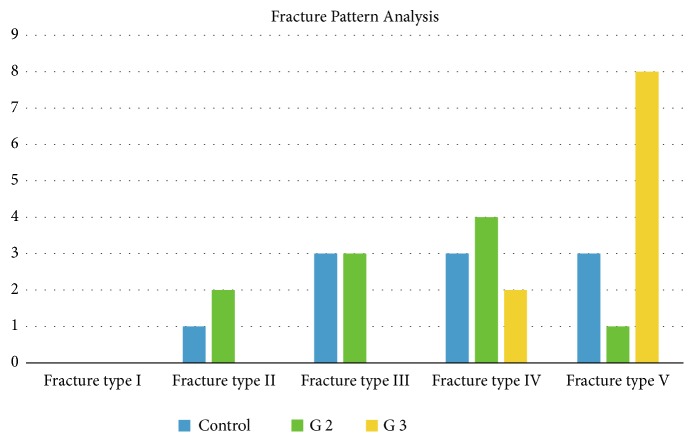
Fracture Pattern classification.

Flaws were classified and evaluated using a magnifying lens and rated as described by Burke et al. [6]: (I) minimal fracture or crack in the crown; (II) less than half of the lost crown; (III) fracture of the crown through the midline, half-crown displaced or lost; (IV) more than half of the lost crown; and (V) severe fracture of tooth and/or crown.  Figure 9 shows the fracture pattern of the groups.

## 4. Discussion

The results of this study confirmed the hypothesis that the composite resin build-up of the preparation in human molars, prior to the fabrication of ceramic inlays, did not promote a decrease in the strength of the tooth/inlay assembly with respect to its compressive strength, when compared to cases where the preparations did not require composite resin build-ups. On the other hand, both tested groups (G2 and G3) showed to be significantly less resistant than the control group (healthy tooth). These results corroborate those found by a previous study [18] who researched the compressive strength of premolars restored with ceramic and composite inlays and found that none of the tested groups showed statistically significant differences under compression, except for the control group, which showed statistically significant higher values.

Regarding the pattern of fracture, assessed on a percentage basis, it can be noted that most of the failures observed in Group 3 were type V, with severe coronal destructions, and more aggressive when compared to the group with support only on tooth structure (G2). However, even if there is this classification, it should be considered that these failures occurred in rather higher forces (MPa) than the ones that the tooth is clinically exposed to. Thus, it should be taken into account that even though Group 3 showed a higher concentration of severe failures, this approach is still a therapeutic alternative.

Unlike the results found in this study, Saridag et al. [19] showed no statistically significance difference between the groups tested (inlays and control). In clinical situations where the removal of carious tissue or a defective restoration produced irregularities in the pulpar wall, the present study research showed that these irregularities or even the loss of the whole pulp wall might be rebuilt or filled with composite resin. In this study it was found that the preparation of the composite build-up did not reduce the strength of the tooth/restoration assembly when compared to preparation supported only by tooth structure.

Using composite resin enables the adjustment of the walls and gives an appropriate and uniform depth of the preparation, along with providing an ideal thickness for the ceramic material, with a proper radiopacity without interfering with the final shade of the indirect restoration [20].

In this study, the occlusal isthmus of the preparation was standardized in two thirds of the intercuspal labiolingual distance while the opening of the proximal boxes was standardized in two thirds of the buccolingual distance measured from the prosthetic equator, unlike other studies, in which standardized preparations were carried out, ignoring the labial-lingual dimension of each tooth [15, 19, 21, 22]. On the other hand, several studies highlight the importance of material thickness, noting that it may vary from 1.5 to 2.0 mm [12]. It has been argued that the depth/thickness should be between 1.2 mm and 1.5 mm to ensure strength and decrease the risk of fractures of ceramic restorations in posterior teeth, while the reduction the proximal regions should be between 1.2 mm and 2 mm.

It has been found that industrially manufactured partial ceramic crowns should have at least 1.5 – 2.0 mm thickness in areas subjected to stress, in order to prevent crack formation and covery long-term fracture resistance of ceramic [12]. In other words, the risk of failure is minimized in inlays by increasing the minimal thickness of ceramic [13]. As the occlusal contact occurs naturally near the margins, the risk of adhesive failure is increased, leading to increased fracture risk in occlusal surfaces if the ceramic thickness is lower than the specified.

It is important to understand the composition and mechanical properties of dentin in order to understand how to dissipate the masticatory forces distributed throughout the tooth, along with allowing a prediction of how these forces may cause changes in the restorations. The compressive strength of dentin varies from 230 to 370 MPa, and its modulus of elasticity varies from 7 to 30 GPa [23, 24]. In addition, the modulus of elasticity of dentin was found to be 18 GPa and its compressive strength of 297 MPa [7]. Composite resins have the ability of transferring stresses under compressive forces. These stresses are transferred from the matrix to the filler particles, similarly to the organic part of dentin and its mineral content. Composite resins present elasticity modulus between 3 and 6 GPa and their compressive strength ranges about 75 MPa [7].

Building-up the preparation with composite resin also facilitates image acquisition by the CAD/CAM system and reduces heat transfer to the pulp tissue. All-ceramic restorations became widespread in the dental market in the 1980s, when the ceramic reinforced by leucite and the possibility of them being conditioned with acids and cemented to the tooth structure was introduced. The material properties regarding strength and aesthetics were also improved, making a more reliable ceramic material [25, 26].

In the same decade, CAD/CAM systems have been introduced and have been gradually expanding until today. Conventional impressions may now be replaced by image acquisition of the preparation using a scanner that allows designing and fabricating a restoration in the same appointment, saving time and avoiding clinical distortions, which are very common in the conventional protocols, producing restorations with good marginal adaptation, color stability and high success rates [27–29]. Another great advantage of using CAD/CAM techniques compared to direct resin composites is the lower polymerization shrinkage (which is limited to the shrinkage from curing of the resin cement), reducing the size of the gaps at the tooth/restoration interface [30–32]. Both tested groups were restored with lithium disilicate-based ceramic, which have advantages such as its superior flexural strength (350 MPa) and fracture toughness (3.2 MPa.m^0,5^) [33]. Its microstructure is composed of approximately 60% lithium disilicate crystals (0,5-5*μ*m extension) dispersed in a glass matrix. These characteristics make it etchable with hydrofluoric acid, providing micromorphologic changes that act like microretentions for adhesive bonding to the tooth structure.

Holberg et al. [34] performed a finite element analysis assessing the risk of fracture of lithium disslicate ceramic inlays with different thicknesses and variations of angle, depth, and width of the preparations and showed that the reduction in the ceramic thickness of the inlays does not imply in higher risk of fractures. In conclusion, when the samples are under normal occlusal force, the inlay volume was not an important factor influencing tensile stresses, so it can support the argument that all-ceramic inlays do not have an increased risk of fracture and are suitable for minimally invasive treatment.

In a 9-year-clinical follow-up study that evaluated the clinical outcomes of crowns made with lithium disilicate after 9 years of cementation the cumulative rate of 97.4% survival rate at 5 years and 94.8% after 8 years of clinical function was found [35]. The completion of the compression test did not take place immediately after the cementation in order to allow the subsequent polymerization of the resin material [36]. It is known that the size of the device for compressive testing as well as the speed influences the results. In this study, a speed of 1 mm/min of the metal ball with 6 mm in diameter was used until the fracture. The speed rate of studies evaluating the compressive strength in premolars ranges from 0.02 mm/min [37] to 1 mm/min [19], mostly between 0.5 mm/min [19, 38] and 1 mm/min [20]. Because this study was performed in molars, which bears maximal compressive strengths in the oral environment, the 1 mm/min speed until failure was used.

There is no consensus in the literature regarding the location where the compressive forces should be applied, whether in the areas of cusp, at tooth/restoration interface, or merely on the restoration. In this study, the force was applied at the tooth/restoration interface, similar to previous studies, [37, 38] because after cementation the tooth and the restoration behave like a single unit. This was the contrary of studies that applied the compressive force at the occlusal surfaces, touching only the restorative material [19, 34, 39].

A previous study [40] that evaluated by means of the frequency of sound vibration, the maximum occlusion force during mastication and swallowing found maximum strength in 20 subjects evaluated during mastication of 26.7 Kg and during swallowing stage found maximum force of 30.2 kg. Comparing the maximum forces with the results found in this study, if the results are converted to MPa KgF/mm^2^ the control group had an average compressive strength of 331.07 KgF/mm^2^, group 2: 201.05 KgF/mm^2^, and group 3: 174.1 KgF/mm^2^. Therefore, even if the groups tested have shown a resistance to compressive loads significantly lower than the healthy tooth, these values are still raised to the maximum force of masticatory load. This means that teeth restored with ceramic inlays (G2 and G3) have sufficient strength to withstand the masticatory loads.

Considering the limitations of this study, it can be concluded that MOD cavity preparations with similar configuration do not present statistically significant differences after reconstruction with composite resin and dentin support, when subjected to compressive load. Also, the tested groups restored with lithium dissilicate did not reestablish the healthy tooth resistance yet provided sufficient fracture resistance to withstand physiological masticatory forces.

## Figures and Tables

**Table 1:** Mean compressive strength (N) obtained by the control group, G2, and G3 and their descriptive statistics (standard deviation, minimum, maximum, coefficient of variation, and 95% confidence interval).

Groups	N	Average (Newtons)	Standard Deviation	Minimum	Maximum	CV	-IC(95%)	+IC(95%)
Control	10	3276.14	623.52	2317.44	4092.60	197.17	2830.10	3722.19

G2	10	1971.71	557.01	1322.57	2987.35	176.14	1573.25	2370.18

G3	10	1707.82	436.30	1270.14	4092.60	137.97	1395.71	2019.94

**Table 2:** Test of normality.

Shapiro-Wilk's			
Groups	Statistic	Df	Sig.
Control	,880	10	,131

G2	,884	10	,144

G3	,892	10	,179

**Table 3:** Levene's homogeneity of variances test.

Levene's statistics	df1	df2	Sig.
1,199	2	27	0,317

**Table 4:** One-way analysis of variance test (ANOVA).

	Sum square	df	Mean square	F	Sig.
Between groups	14102706.97	2	7051353.48	23.784	0.000001

Within groups	8004767.34	27	296472.86		

Total	22107474.31	29			

**Table 5:** Average compressive strength values of each group and their respective standard deviations (SD).

Groups	Mean (±SD)
Control	3276.14 (±623.52)a

G2	1971.71 (±557.01)b

G3	1707.82 (±436.30)b

Mean followed by the same letters indicates no statistically significant differences between the groups according to Tukey's post hoc at a significance level of 0.05.

## Data Availability

The data used to support the findings of this study are included within the article.
